# Sex differences in cause-specific mortality: regional trends in seven European countries, 1996–2019

**DOI:** 10.1093/eurpub/ckad111

**Published:** 2023-07-28

**Authors:** Markus Sauerberg, Sebastian Klüsener, Michael Mühlichen, Pavel Grigoriev

**Affiliations:** Federal Institute for Population Research (BiB), Wiesbaden, Germany; Federal Institute for Population Research (BiB), Wiesbaden, Germany; Vytautas Magnus University, Kaunas, Lithuania; University of Cologne, Cologne, Germany; Federal Institute for Population Research (BiB), Wiesbaden, Germany; Federal Institute for Population Research (BiB), Wiesbaden, Germany

## Abstract

**Background:**

Male excess mortality is mostly related to non-biological factors, and is thus of high social- and health-policy concern. Previous research has mainly focused on national patterns, while subnational disparities have been less in the focus. This study takes a spatial perspective on subnational patterns, covering seven European countries at the crossroad between Eastern and Western Europe.

**Methods:**

We analyze a newly gathered spatially detailed data resource comprising 228 regions with well-established demographic methods to assess the contribution of specific causes of death to the evolution of sex mortality differentials (SMDs) since the mid-1990s.

**Results:**

Our results show that declines in SMDs were mostly driven by a reduction of male excess mortality from cardiovascular diseases and neoplasms (about 50–60% and 20–30%, respectively). In Western Europe, trends in deaths from neoplasms contributed more to the reduction of SMDs, while among regions located in Eastern-Central Europe narrowing SMDs were mostly driven by changes in cardiovascular disease-related deaths. Moreover, men show up to three times higher mortality levels from external causes as compared to women in several analyzed regions. But in absolute terms, external deaths play only a minor role in explaining SMDs due to their small contribution to overall mortality.

**Conclusions:**

We conclude that examining the regional development of SMDs is useful for introducing targeted social and health policies in order to reduce and prevent mortality inequalities between women and men.

## Additional content

An author video to accompany this article is available at: https://oup.cloud.panopto.eu/Panopto/Pages/Viewer.aspx?id=8ac46113-e044-4836-9ad0-b03c00de7ee1.

## Introduction

Ensuring long healthy lives for all at all ages is a major political goal of the European Union (EU), which has allocated resources via the EU cohesion policy plan to assist less developed European regions in narrowing the persisting economic, social and territorial disparities. Social inequalities in health limit the opportunities for disadvantaged population groups to live productive and fulfilling lives. This situation is not only unfair, but also presents substantial challenges to the social welfare state.^1^ The impact of genetics on mortality is comparatively small, suggesting that a large part of the observed inequalities is not inevitable and can be addressed through changes in cultural norms, socioeconomic conditions, lifestyles and public health policies.^2–4^ In this respect, social and regional disparities in mortality are of great concern to social and health policymakers.^5^ Identifying and addressing the underlying causes of these disparities would enable vulnerable groups or regions to catch up to the vanguard populations that have already achieved lower mortality levels.

The chance of reaching old age depends on several factors, including individual’s lifestyle in terms of health behaviour, exposure to mortality risks and access to healthcare.^2^^,^^4^^,^^5^ Consequently, the survival advantage of women at the population level has been primarily attributed to behavioural factors, while biological differences between the sexes play a relatively minor role.^6^^,^^7^ This is why the magnitude of the SMD is not fixed but varies significantly over time and across regions, reflecting changes in social and economic conditions.^8^^,^^9^ In this sense, large SMDs can be viewed as an indication of unfavourable socioeconomic conditions within a population.

In most European countries and other high-income nations, SMDs were widening during the 20th century. However, since the 1980s and 1990s, SMDs have been showing a narrowing trend throughout Europe.^9–11^ The decline first began in Northern Europe, followed by most western European countries and eventually, SMDs also started to decrease in Eastern Europe.^12^ The evolution of SMDs is linked to differences in the smoking history between women and men, improvements in health care and medical innovations that affected women and men to varying degrees, along with changing gender roles.^13–15^ Eastern Europe experienced a later reduction in SMDs due to various reasons. For instance, the post-communist transition from planned to market economy, along with its economic and social upheavals, placed populations in Eastern Europe under great psychosocial stress.^16^^,^^17^ This had particularly negative effects on male mortality levels and further increased SMDs.^18^^,^^19^

While many studies have focused on national SMD patterns,^8–11^ there is limited knowledge about regional variations in the development of SMDs. The few existing studies, however, have found substantial differences between regions, suggesting that figures based on national statistics may overlook important disparities.^2^^,^^3^^,^^20^^–^^22^ Furthermore, it is crucial to consider causes of deaths as it allows for linking observed mortality patterns to specific lifestyles and risk factors.

We utilize a unique data source that includes cause-specific mortality data from seven European countries (Austria, Denmark, Czechia, France, Germany, Slovakia and Switzerland), divided into 228 spatial units, to analyze the sex gap in an interesting area situated at the intersection of the east-west and north–south mortality gradients characterizing Europe’s spatial mortality pattern.^23^ The study period from 1996 to 2019 is particularly noteworthy due to the aforementioned transition of post-communist countries and the eastward expansion of the European Union.

## Methods

### Administrative data by regions

We rely on official mortality data by age, sex, region, cause of death and corresponding population counts. To achieve a regional classification that is most comparable in size and structure for all seven countries, we aggregated these counts to 228 spatial units, relying mostly on the NUTS-2 level, except where noted: 9 ‘Bundesländer’ for Austria, 8 ‘oblasti’ for Czechia, 5 ‘regioner’ for Denmark, 95 ‘départements’ for France (NUTS-3), 96 ‘Raumordnungsregionen’ for Germany (no NUTS level), 8 ‘kraje’ for Slovakia (NUTS-3) and 7 ‘Grossregionen’ for Switzerland.

### Grouping of causes of deaths

We split death counts into five cause-of-death groups (see Supplementary table S1). The first group includes cardiovascular diseases (CVD). Deaths from cancer of larynx, trachea, bronchus and lung are assigned to the second group, shortly referred to as lung cancer. All remaining deaths from neoplasms are included in the third group. We decided to look at lung cancer deaths separately due to their link to past smoking behaviour.^24^ It should be noted, however, that smoking also affects the level of CVD and other neoplasms. The fourth group refers to external causes, such as accidents, assaults and suicides, as well as deaths from alcohol and drug abuse. The last group includes the remaining causes. To improve data comparability over time and between the countries, we redistributed unknown and ill-defined deaths by year, region, age and sex across well-defined causes by means of proportional redistribution, i.e. proportionally to the share in the total number of deaths.

### Statistical analysis

We calculated age-specific death rates covering the age intervals, 0–9, 10–19, 20–24, 25–29, …, 80–84, 85+. To avoid random fluctuations due to small death counts, we aggregated deaths and exposures (defined as mid-year population counts) over a 3-year period (1996–98, 1999–2001, …, 2017–19) and smoothed the data by applying the R function call ‘Mort1Dsmooth (Age, Deaths, log(Exposures), method = 4, df = 5)’ using the MortalitySmooth package.^25^ Those smoothed age-specific death rates were used to obtain standardized death rates (SDRs) based on the European Standard Population 2013. We rely on SDRs instead of the period life expectancy indicator as a summary measure of mortality because SDRs are a linear function of age-specific death rates, which makes them extremely convenient from a mathematical point of view.^26^

For France, the most recent calendar year with available mortality data was 2017. Consequently, SDRs for the last period in France do not refer to three calendar years (2017–19) but only to 1 year. Cause-specific death counts for France were only available in 10-year age intervals. We ungrouped and re-aggregated age-specific death counts by cause using the well-established ‘pclm’ function from the ‘ungroup’ R package in order to derive SDRs based on the same age intervals as for the other countries.^27^^,^^28^

Moreover, we rely on a regression-based approach for estimating the cause-specific contributions to the change in SMDs over time. This method was pioneered by Preston^29^ and has been used for examining sex differences in mortality previously.^30^ The difference in the cause-specific SDR between men and women is modelled as a linear function of the overall difference in the SDR between men and women,


$${\mathrm{SDR}}_{\mathrm{Men}}\left(c,i,t\right)-{\mathrm{SDR}}_{\mathrm{Women}}\left(c,i,t\right)=a_{i}+b_{i}\left({\mathrm{SDR}}_{\mathrm{Men}}\left(i,t\right)-{\mathrm{SDR}}_{\mathrm{Women}}\left(i,t\right)\right),$$

where $\mathrm{SD}R_{\mathrm{Men}}(c,i,t)$ and $\mathrm{SD}R_{\mathrm{Women}}(c,i,t)$ correspond to the SDR for cause *c* in time *t* and region *i* for women and men, respectively. In each region, the sum of the $b_{i}$ coefficients across all causes equals one. Thus, $b_{i}$ can be interpreted as the proportionate change in the overall difference in the SDR between women and men that is attributable to a change in the SDR sex difference for cause *c*. The time series of cause- and region-specific SDRs can be found in the Supplementary material.

## Results

### Time trends in all-cause SMDs by regions

Table 1 presents the quartiles of region-specific SMDs (in absolute and relative terms) for eight periods across broad age groups. Mortality levels are higher for men in all analyzed regions, but the gap has been continuously decreasing over time. This trend holds true for all age groups, regardless of whether SMDs are measured in absolute or relative terms. For instance, the median value for absolute differences in SDRs between the two sexes decreased from 608 to 360 between the periods 1996–98 and 2017–19, while the median statistic for relative differences (SDR ratio) dropped from 1.70 to 1.59 over the same time span.

**Table 1:** Summary statistics on regional SMDs by period and age group (*N*=228 spatial units)

Period	Sex difference (men–women)					SDR ratio (men/women)				
All ages	Min.	*Q*_1_	Median	*Q*_3_	Max.	Min.	*Q*_1_	Median	*Q*_3_	Max.
1996–98	467	563	608	672	971	1.51	1.65	1.70	1.85	2.06
1999–2001	415	517	556	606	951	1.47	1.62	1.69	1.85	2.00
2002–04	351	467	511	567	894	1.44	1.58	1.65	1.78	1.95
2005–07	312	415	458	509	896	1.42	1.56	1.65	1.79	1.98
2008–10	289	380	423	472	823	1.36	1.54	1.64	1.77	1.91
2011–13	250	350	392	450	752	1.41	1.52	1.63	1.75	1.95
2014–16	242	334	371	427	719	1.39	1.50	1.62	1.73	1.87
2017–19	214	317	360	407	658	1.38	1.48	1.59	1.70	1.92
Age 0–39										
1996–98	31	45	57	70	109	1.55	1.98	2.17	2.46	5.55
1999–2001	27	41	51	64	97	1.55	1.95	2.17	2.45	5.33
2002–04	22	36	47	60	95	1.50	1.93	2.16	2.53	6.28
2005–07	14	32	42	53	100	1.43	1.87	2.12	2.53	8.10
2008–10	14	28	36	50	79	1.43	1.84	2.12	2.56	5.57
2011–13	9	24	32	43	71	1.38	1.78	2.07	2.48	6.77
2014–16	0	22	29	39	73	1.03	1.74	1.96	2.39	7.01
2017–19	3	20	26	35	114	1.14	1.69	1.88	2.27	9.82
Age 40–64										
1996–98	207	359	416	500	996	1.43	2.08	2.28	2.46	3.08
1999–2001	188	326	381	446	1000	1.42	2.02	2.25	2.44	2.98
2002–04	174	301	350	418	929	1.52	1.98	2.15	2.36	2.98
2005–07	153	272	324	386	832	1.55	1.96	2.13	2.31	3.00
2008–10	145	253	303	367	809	1.49	1.93	2.07	2.27	3.01
2011–13	131	228	280	341	658	1.56	1.83	2.03	2.23	2.95
2014–16	124	214	257	319	625	1.54	1.79	1.98	2.19	2.76
2017–19	106	195	243	308	588	1.52	1.76	1.90	2.15	3.37
Age 65+										
1996–98	1727	2193	2385	2532	3236	1.42	1.55	1.61	1.73	1.90
1999–2001	1640	2014	2157	2331	3152	1.40	1.54	1.60	1.71	1.84
2002–04	1420	1835	1951	2143	2967	1.38	1.50	1.55	1.67	1.80
2005–07	1212	1595	1754	1907	3089	1.37	1.48	1.54	1.68	1.82
2008–10	1128	1468	1623	1793	2890	1.33	1.46	1.53	1.65	1.79
2011–13	1003	1375	1504	1682	2733	1.35	1.45	1.52	1.64	1.84
2014–16	967	1317	1435	1594	2690	1.35	1.44	1.52	1.63	1.75
2017–19	910	1281	1389	1537	2318	1.34	1.43	1.51	1.59	1.75

Note: Evolution of the male–female differences in the region-specific standardized death rate (SDR) and in the male/female ratio of SDR measured through the observed minimum, the first quartile *Q*_1_ (25% of observed sex differentials are lower than this value), median (half of observed sex differentials are lower than this value) and the third quartile *Q*_3_ (75% of observed sex differentials are lower than this value).

The largest relative differences are observed among young individuals (age 0–39) and at middleaged individuals (age 40–64), with maximum SDR ratios ranging from 5.33 to 9.82. However, the number of deaths occurring at young ages is comparatively small in absolute terms. Therefore, the absolute difference in SDRs between women and men is more pronounced for age groups with a higher density of deaths, specifically at ages 65 and older. In other words, mortality is comparatively low at younger ages, but among those who die, a disproportionate number are male.

**Figure 1:**
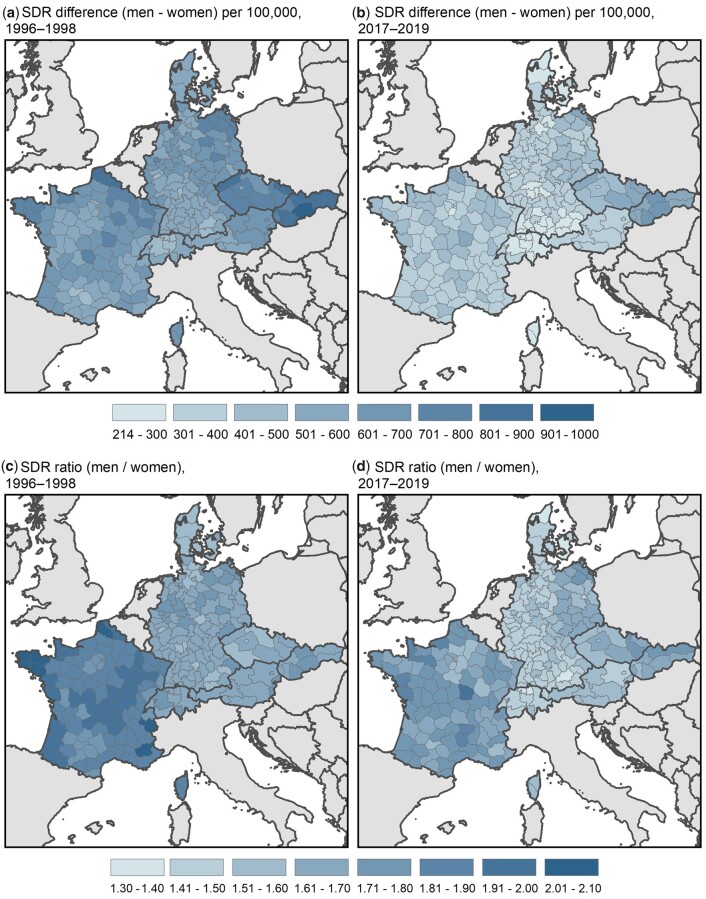
SMDs in 1996–98 and 2017–19 in seven European countries; absolute (a and b) and relative (c and d) differences between men and women in SDRs

Although SMDs have been narrowing over time, considerable regional variation in the magnitude of women’s advantage in survival remains (figure 1). In general, the largest SMDs are observed in Czechia, Slovakia, eastern Germany and France. However, this gap is smaller in capital regions such as Prague or Paris. In eastern Germany and France, regions located in the north exhibit particularly large SMDs. The lowest SMDs are found in Denmark, southern Germany and Switzerland.

### Spatiotemporal trends in cause-specific mortality

**Figure 2:**
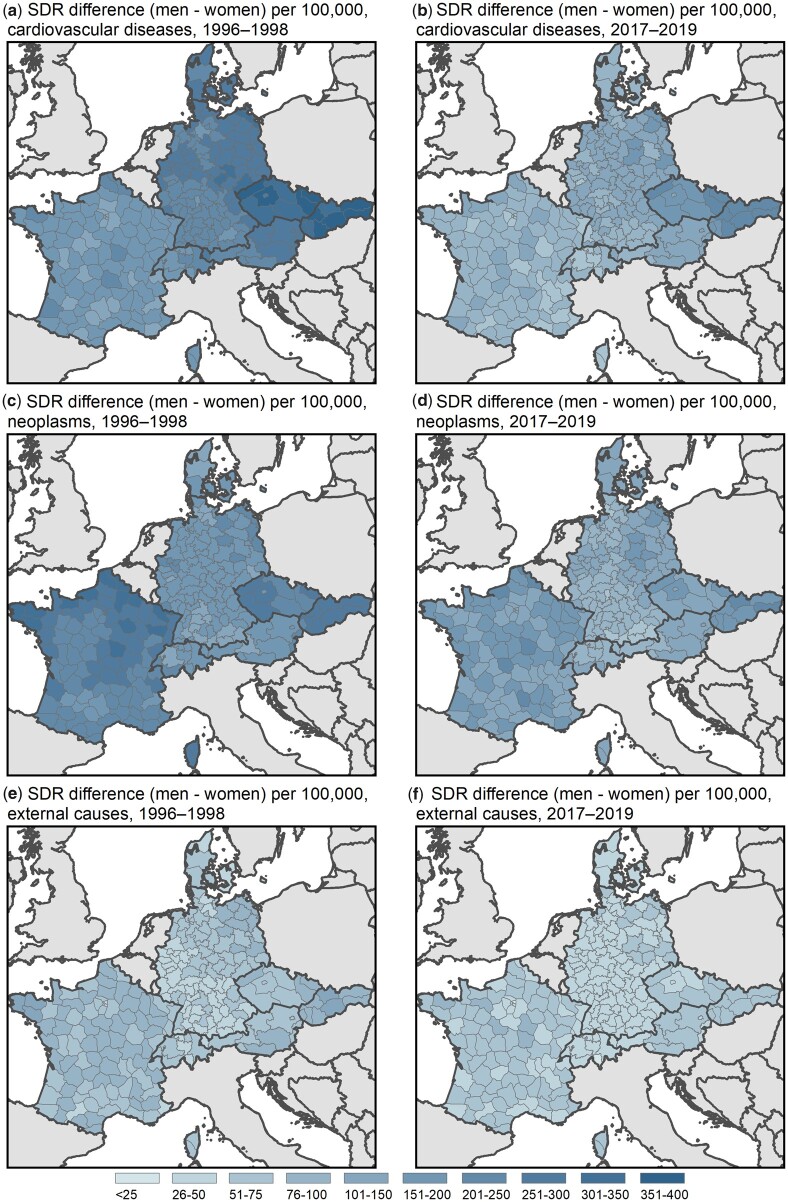
SMDs in 1996–98 and 2017–19 in seven European countries by cause of death; absolute differences (men–women) in SDRs (per 100 000)

Figure 2 presents the absolute differences in cause-specific mortality between women and men, while the corresponding relative differences can be found in the Supplementary material. Trends in absolute differences clearly indicate that SMDs have narrowed for all four selected causes of death over the studied period. For example, male excess deaths from CVD decreased from about 150 to 400 deaths per 100 000 in 1996–98 to about 50–250 deaths per 100 000 in 2017–19. Additionally, the mortality map suggests a clear geographical pattern, with larger sex differentials in CVD-related mortality observed in Central-Eastern Europe. In particular, regions in Slovakia and Czechia show large sex gaps, whereas the lowest differences between women and men are observed in France and Switzerland. Germany and Austria exhibit an east-west gradient, which is more pronounced in the earlier period. Moreover, results for Germany indicate that male excess mortality from CVD is larger in the north compared to the south of the country.

Trends in relative differences suggest small reductions in SMDs related to external causes and CVD (see Supplementary figure S1). Male excess deaths from neoplasms show stronger declines over time, with SDR ratios ranging from about 1.5 to 2.5 in 1996–98 and from 1.4 to 2.0 in 2017–19. France shows comparatively large SMDs in neoplasms, similar in size to the sex ratios observed in Slovakia and Czechia. Further, male excess mortality from neoplasms is highest in the north of France (especially in absolute terms). In contrast to sex differentials in CVD-related mortality, Germany’s east-west gradient is more pronounced in the later period compared to 1996–98 (in both absolute and relative terms).

The level of mortality from external causes decreased for both sexes. Consequently, absolute SMDs also narrowed over the observed time span. The relative differences between women and men, however, did not decline between the two periods. In some regions, the SDR ratios for external causes even increased between the two periods. For instance, parts of France and Germany, and Czechia as a whole show larger SDR ratios in 2017–19 as compared to the period 1996–98. SMDs from external causes are most pronounced in Central-Eastern and Central-Western Europe, while regions falling in-between such as Germany (except for the north-eastern part), Denmark, western Austria and Switzerland show the smallest levels of male excess mortality.

### Cause-specific contributions to narrowing SMDs

**Figure 3:**
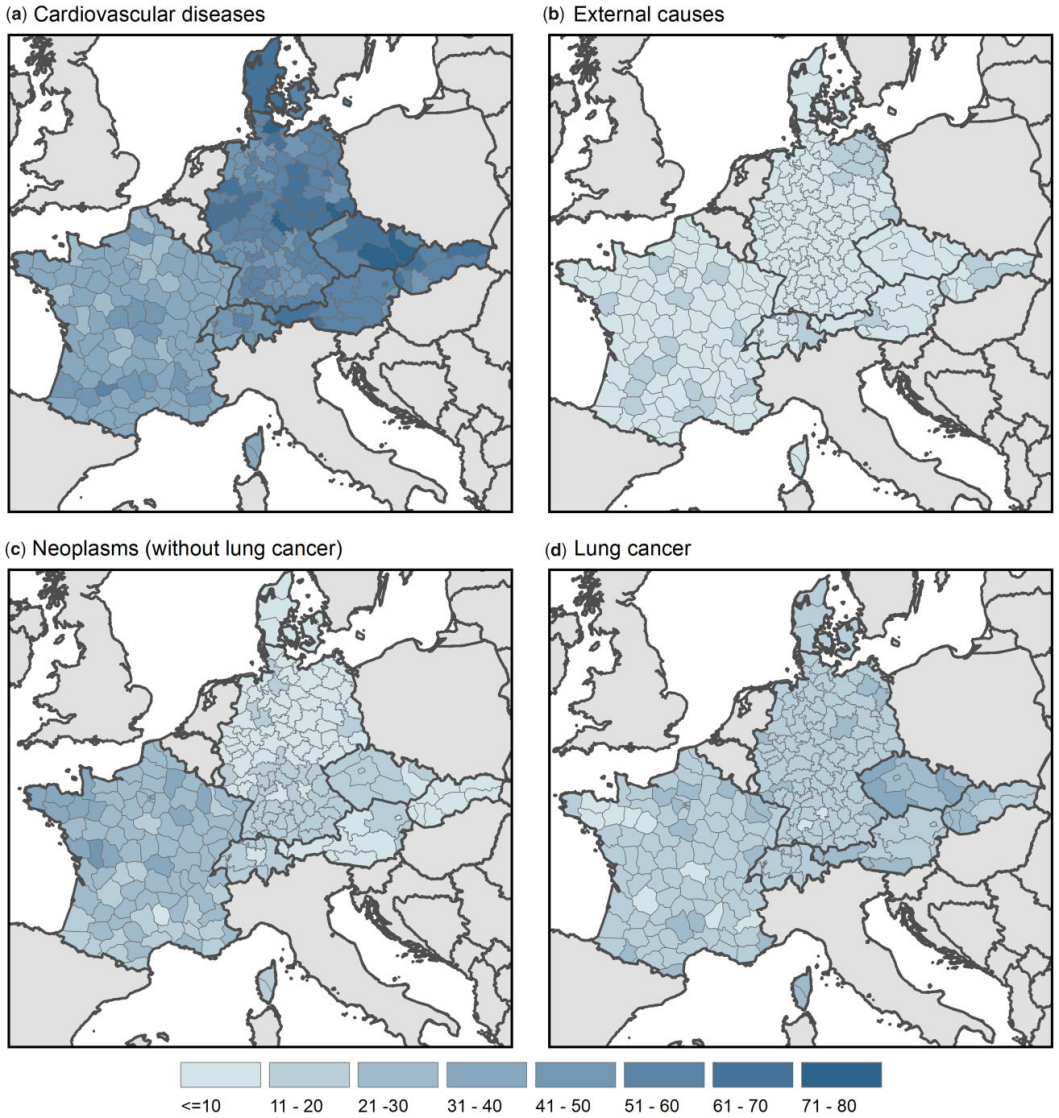
Cause-specific contribution (in percent) to the change in absolute SMDs between 1996–98 and 2017–19 in seven European countries

Figure 3 presents the cause-specific contributions to the change in absolute SMDs between 1996–98 and 2017–19 (in percent). Deaths related to CVD contributed most to the narrowing sex gap in the vast majority of analyzed regions (about 50–80%). However, the contributions are considerably lower in the French regions (about 30–40%). Changes in deaths from external causes had a smaller impact on decreases in SMDs, with their contribution being below 10% for most regions. Yet, in north-eastern Germany, as well as in some regions in Slovakia, Austria, Switzerland and France, the estimated contribution of external causes reaches up to 20%.

In France, changes in cancer-related mortality contributed more to the decrease in the sex gap (about 20–40%) as compared to other countries (10–20%). In most analyzed regions, the impact of lung cancer is larger than the effect of the remaining neoplasms (11–30%). Especially, parts of Czechia exhibit comparatively large contributions from lung cancer to narrowing SMDs (about 30–40%). This is due to a decrease in mortality from lung cancer among men but an increase among women.

We performed a sensitivity analysis using the period life expectancy indicator instead of SDRs and found no substantial differences in our main results, i.e. changes in the number of deaths from CVD and neoplasms have contributed most notably to narrowing SMDs. The corresponding figures, as well as the R code used for this study, can be found in the online repository (https://osf.io/xe8uc/).

## Discussion

While analyses of overall mortality clearly indicate the presence of east-west and north–south gradients across Europe, our examination of SMDs reveals a more nuanced pattern.^17^^,^^23^ In addition to Czechia, Slovakia and eastern Germany, we found large SMDs in France. This is particulary true when measuring SMDs through sex ratios, as France shows the largest relative differences in mortality between women and men. However, capital regions such as Prague and Paris demonstrate smaller sex gaps in mortality. Furthermore, hot spots of male excess mortality are located in northern France and north-eastern Germany, while SMDs are comparatively low in Denmark, southern Germany and Switzerland.

The narrowing of SDMs can be largely attributed to changes in CVD-related mortality, especially in Central- and Central-Eastern Europe. France, on the other hand, deviates from this pattern as the contribution of CVD to the decrease in SDMs is less pronounced. The French population shows relatively low levels of CVD-related deaths, leaving limited room for further improvement. Moreover, changes in deaths from lung cancer have also contributed to the declining differences in mortality between women and men across Europe’s regions, with largest effects observed in Czechia. The estimated contributions of external causes to changes in SMDs are substantially smaller because of their small weight on overall mortality. Yet, relative differences between women and men are exceptionally large for external causes (men showing 2–3 times higher SDRs as compared to women), indicating that those events and diseases have become comparatively rare in absolute numbers but are predominantly occurring in male populations.

Regional disparities in mortality can be ascribed to contextual factors such as inequalities in access to medical infrastructure and diverging health policies, as well as differences in population composition, i.e. individuals of higher socioeconomic status are more likely to reside in advantaged regions and *vice versa*.^31^ Typically, both factors contribute to the observed spatial patterns, and it is difficult to separate one from another.^32^

We find the lowest SMDs in southern Germany (Munich region) and Switzerland. Selective migration likely plays an important role in explaining some of the observed patterns, as structurally strong regions tend to attract people with ‘good risks’ such as higher education. Likewise, structurally weak regions such as north-eastern Germany and northern France which offer fewer job opportunities, may create an unfavourable social environment in terms of social roles, relations and support, thus fostering unhealthy lifestyles, including smoking and alcohol abuse, resulting in higher mortality rates for both women and men. However, previous research suggests that a disadvantaged subpopulation among men experiences disproportionately high mortality levels, thus leading to widening SMDs for those regions.^33^ Accordingly, factors, such as changes in income or employment status should be understood as gender-based differentials that affect men and women to varying degrees. Traditional gender roles, with men as the primary bread-winners, may make them more susceptible to feelings of frustration and resentment, which can lead to high-risk behaviours and unhealthy lifestyle choices.^19^

For instance, higher sex gaps in restructuring areas, such as the Ruhr region in western Germany or Nord-Pas-de-Calais, Yonne and Nièvre in France, may be linked to rising unemployment rates and a larger proportion of unskilled workers with elevated mortality risks.^21^^,^^22^ Changes in the magnitude of the sex gap over time may be caused by changes in place-specific sex differences in contextual factors, such as unemployment rates, educational levels, working conditions, poverty rates and international and internal migration.^34^ Identifying the exact reasons for narrowing sex gaps in specific areas would require case studies to disentangle them, which is beyond the scope of this article.

Moreover, we observe a faster reduction of CVD-related mortality among males because they are catching up to women in the process of the cardiovascular revolution.^35^ Women started at initially lower mortality level and achieved longevity gains earlier than men did. Going beyond national aggregates reveals regional disparities regarding the mortality convergence between women and men. Urban areas, such as Prague and Bratislava, are more advanced in developing their healthcare system as compared to more rural areas, thus appearing more successful in preventing CVD-related deaths.

Understanding the evolution of SMDs across Europe requires considering past smoking behaviour. In general, women experienced the smoking epidemic later than men.^36^ While men have reached their peak in smoking-attributable mortality at about the end of the 1980s, it is still increasing among women in most European countries.^37^ Further, tobacco control measures were implemented at different times throughout Europe’s regions and consequently, the observed levels and trends in regional SMDs depend on women’s and men’s current stage in the smoking epidemic.^38^ It is difficult to separate the impact of past smoking and the progress in the aforementioned cardiovascular revolution on the observed levels and trends in CVD-related mortality. Both mechanisms contribute to narrowing SMDs, and there may be regional variation in the weight of each effect.

The vast majority of deaths occur at older ages, where CVD and neoplasms are the leading causes. Accordingly, we find the strongest effects on narrowing SMDs for these ages and causes. Social policymakers are, however, often interested in levels and trends in premature mortality, as deaths at younger ages are typically avoidable, and large inequalities between population groups can be considered unfair.^1^^,^^39^ Aside from the expected decline in external causes for former communist countries, we also observe comparatively large SMDs in parts of France, Austria and north-western Germany. In certain regions of France, sex ratios have even increased over time for the age group 0–39. This finding suggests unfavourable lifestyle choices with high-risk behaviours and, ultimately, difficulties of social and economic integration for young men in those regions.^21^^,^^22^

As indicated by our findings, looking at absolute or relative mortality differences between women and men can lead to different conclusions about the change in the sex gap over time. In general, absolute figures are valuable for policymakers because they indicate the magnitude of mortality differentials and how the size of the gap has evolved over time. However, one drawback of absolute numbers is their dependence on the underlying mortality level, i.e. subtracting two small numbers will necessarily result in another small number, which is not the case for relative differences. In practice, reducing mortality differentials in relative terms becomes extremely difficult whenever overall death rates are declining over time. This is because achieving larger relative reductions in mortality for one population group compared to another would be required, which is rarely observed empirically.^40^

### Strengths and limitations

To the best of our knowledge, this is the most comprehensive study on regional variation in SMDs across Europe so far. We examined 228 regions in seven neighbouring countries, covering Northern Europe (Denmark), Western Europe (France) and Central Europe (Austria, Czechia, Germany, Slovakia and Switzerland) over a time span of two decades. Looking at cause-specific mortality on the regional level is challenging due to the stochasticity resulting from small death counts. We selected four broad groups in order to address reliability and comparability over time and space. However, this procedure prevents separating the effects of specific diseases.

## Conclusion

Overall, our study reveals that male excess mortality is highest in structurally disadvantaged regions and lowest in areas with favourable economic conditions. Selective migration, differences in the population composition, and contextual factors, such as access to healthcare, are key drivers of the spatial distribution of SMDs. While national statistics on SMDs suggest a clear east-west gradient, our results unveil a considerably more nuanced pattern. Furthermore, our analysis of cause-specific mortality highlights that SMDs are influenced not only by CVD-related mortality but also by neoplasms, particularly in French regions where mortality levels are mostly driven by this cause. Finally, capital regions in former communist countries seem to be further progressed in reducing initially high mortality levels among men as compared to rural areas. Selective migration may have also contributed to the lower sex gap in post-communist capitals. These findings underscore the importance of implementing targeted social and health policies aimed at reducing and preventing mortality inequalities between women and men.

## Supplementary Material

ckad111_Supplementary_Data

## Data Availability

The data underlying this article cannot be shared publicly due to data protection reasons. Data will be shared on request to the corresponding author with permission of the statistical offices. In the case of Germany, we accessed data through the Research Data Centre (https://www.forschungsdatenzentrum.de/en) in Wiesbaden, Germany. The summary measures of mortality presented in this study and the corresponding R code are available at: https://osf.io/xe8uc/. For the first time, we examine (i) whether there are regional differences across European countries in the development of sex mortality differentials (SMDs) and (ii) whether the cause-specific contributions to narrowing SMDs differ across regions. Urban areas in Central-Eastern Europe are more advanced in reducing excess male mortality as compared to more rural areas, whereas structurally weak regions in both Western and Eastern Europe show larger sex gaps than regions with a favourable economic situation. Reducing mortality from cardiovascular diseases contributed most to narrowing SMDs for the vast majority of analyzed regions. A substantial share of male excess mortality can be avoided through targeted health policies that reduce the risks of dying from cardiovascular diseases and neoplasms.
