# Supplementary material for: Sex differences in cause-specific mortality: regional trends in seven European countries, 1996–2019
Source: Eur J Public Health. 2023 Jul 28;33(6):1052–9. doi: 10.1093/eurpub/ckad111 (PMC10710349; doi:10.1093/eurpub/ckad111)
Supplement: ckad111_Supplementary_Data [file ckad111_supplementary_data.pdf]

Sex Differences in Cause-Specific Mortality: Regional Trends in Seven European Countries, 1996–2019

SUPPLEMENTAL MATERIAL

1. Information on causes of death groups and corresponding ICD codes (Table S1)
2. Sex mortality differentials by causes of death, relative differences (Figure S1)
3. Sex mortality differentials by age group, absolute and relative terms (Figures S2–S4)
4. Standardised death rates by sex and age group (Figures S5–S8)
5. Regional time trends in standardised death rates by sex and cause of death (Figure S9)

Table S1 Causes of death used in the analysis according to ICD-9 and ICD-10 codes

Cause-of-death group	ICD-9 codes	ICD-10 codes
Cardiovascular diseases	390–398, 401–405, 410–459	I00–I15, I20–I52, I60–I99, G45
Lung cancer	161–162	C32–C34
Neoplasms (without lung cancer)	140–160, 163–239	C00–C31, C37–D48
External causes (incl. alcohol- and drug-related deaths)	E800–E949, E950–E999, 291, 303–305	V01–V99, W00–W99, X00–Y36, F10–F19
Remaining causes of death	Remainder	Remainder

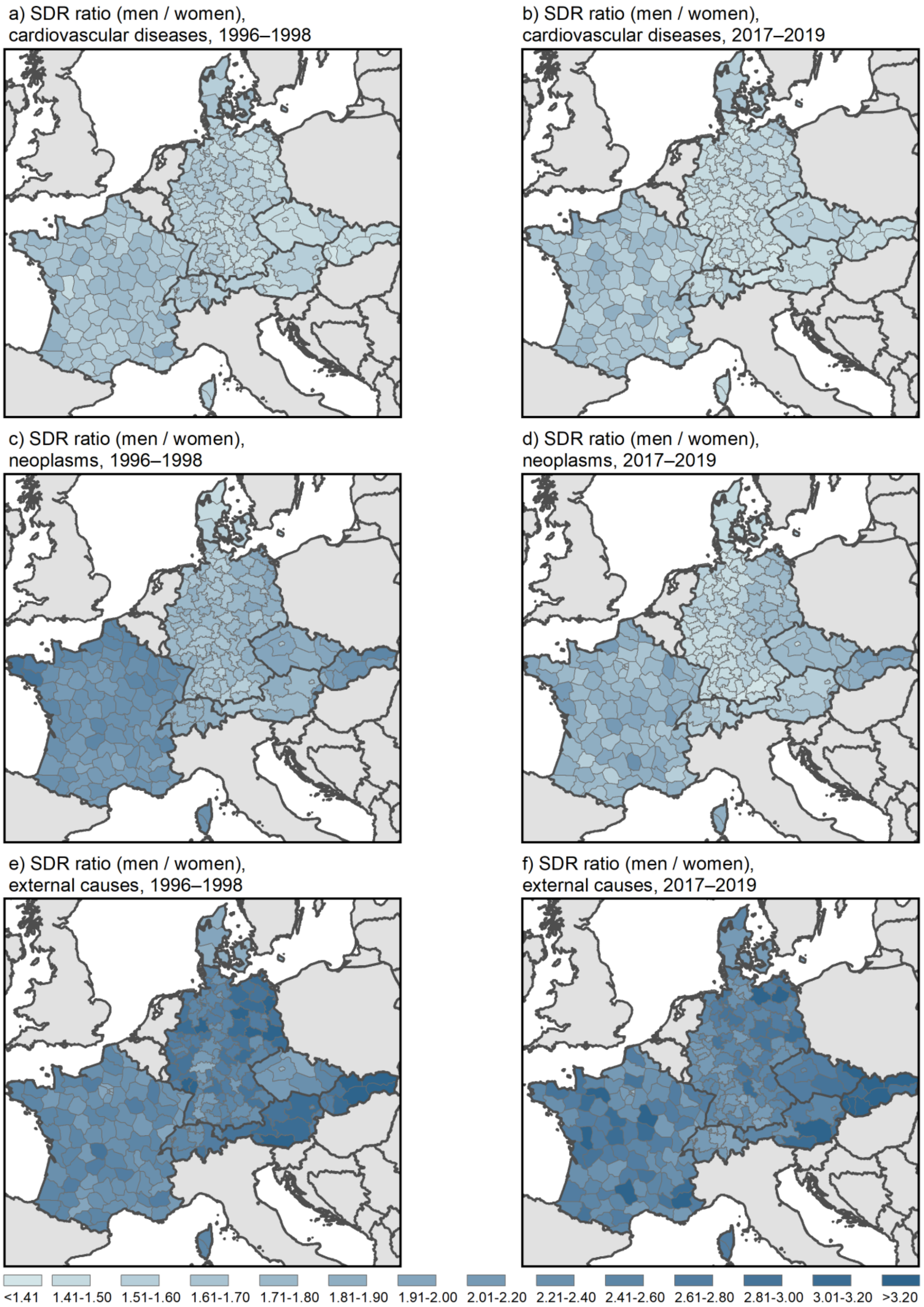

Figure S1 Sex mortality differentials in 1996–1998 and 2017–2019 in seven European countries by cause of death; relative differences (men / women) in standardised death rates

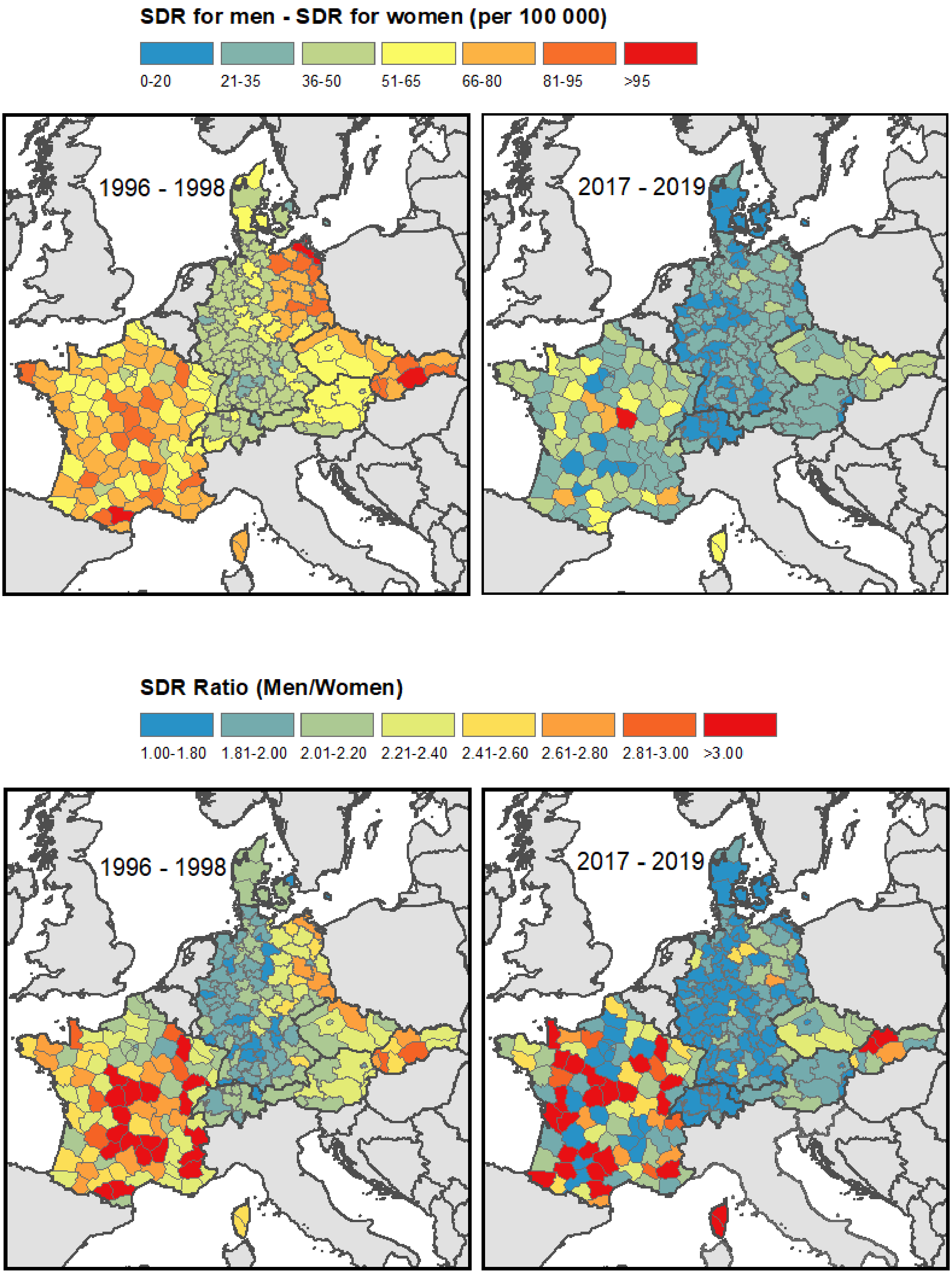

Figure S2 Sex mortality differentials in 1996–1998 and 2017–2019 in seven European countries; absolute (top) and relative (bottom) differences between men and women in standardised death rates (SDR); ages 0–39

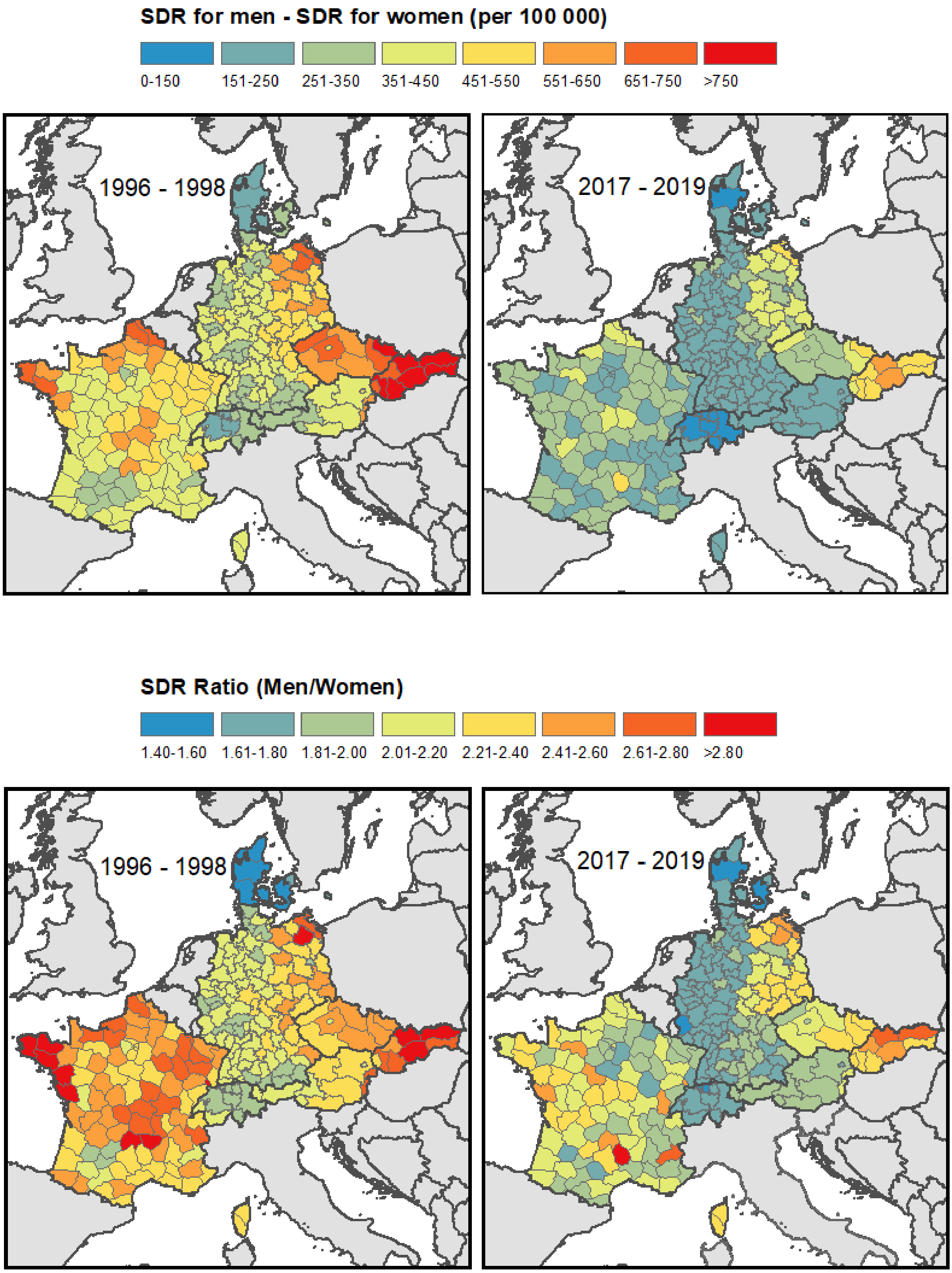

Figure S3 Sex mortality differentials in 1996–1998 and 2017–2019 in seven European countries; absolute (top) and relative (bottom) differences between men and women in standardised death rates (SDR); ages 40–65

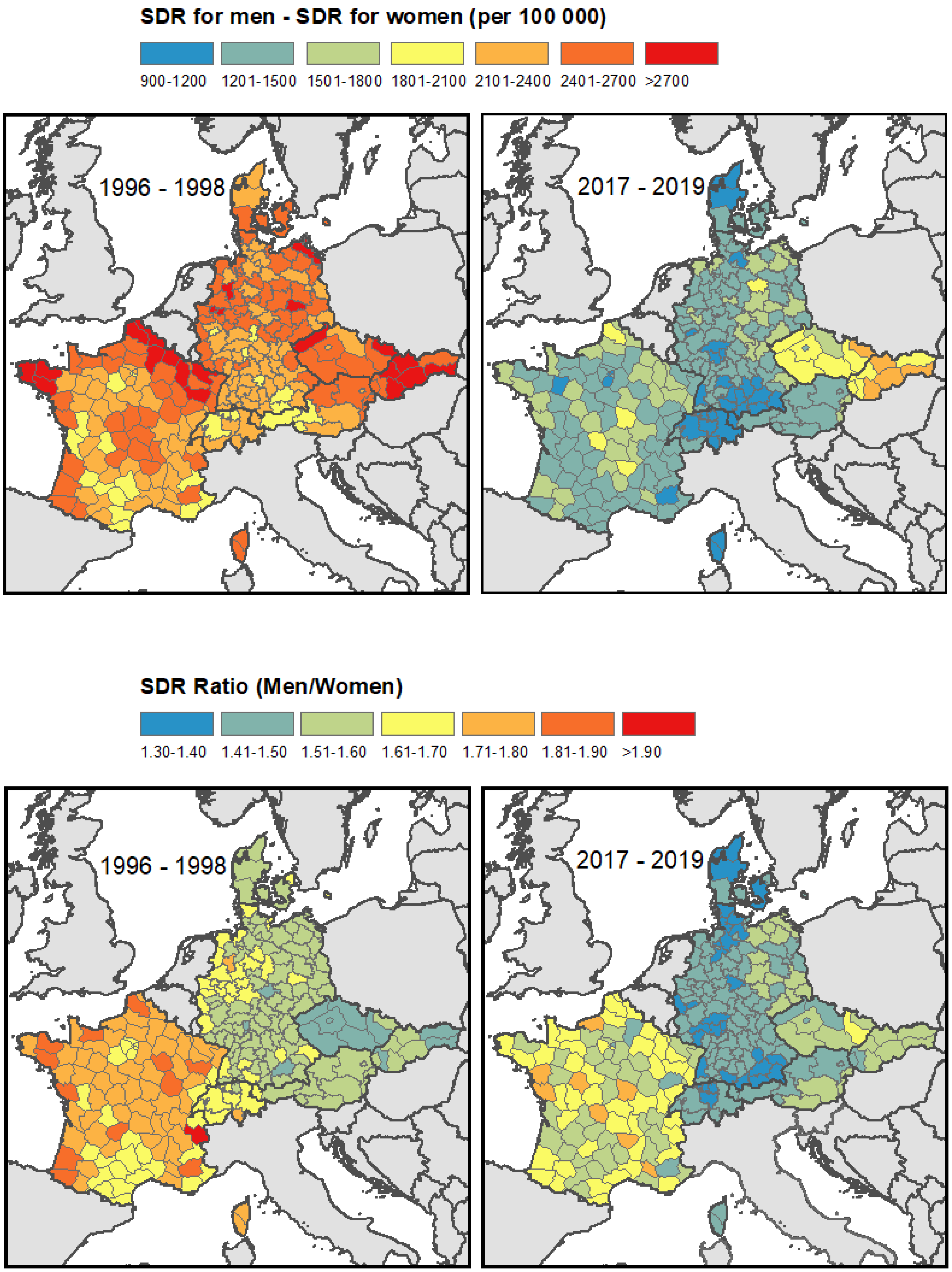

Figure S4 Sex mortality differentials in 1996–1998 and 2017–2019 in seven European countries; absolute (top) and relative (bottom) differences between men and women in standardised death rates (SDR); ages 65+

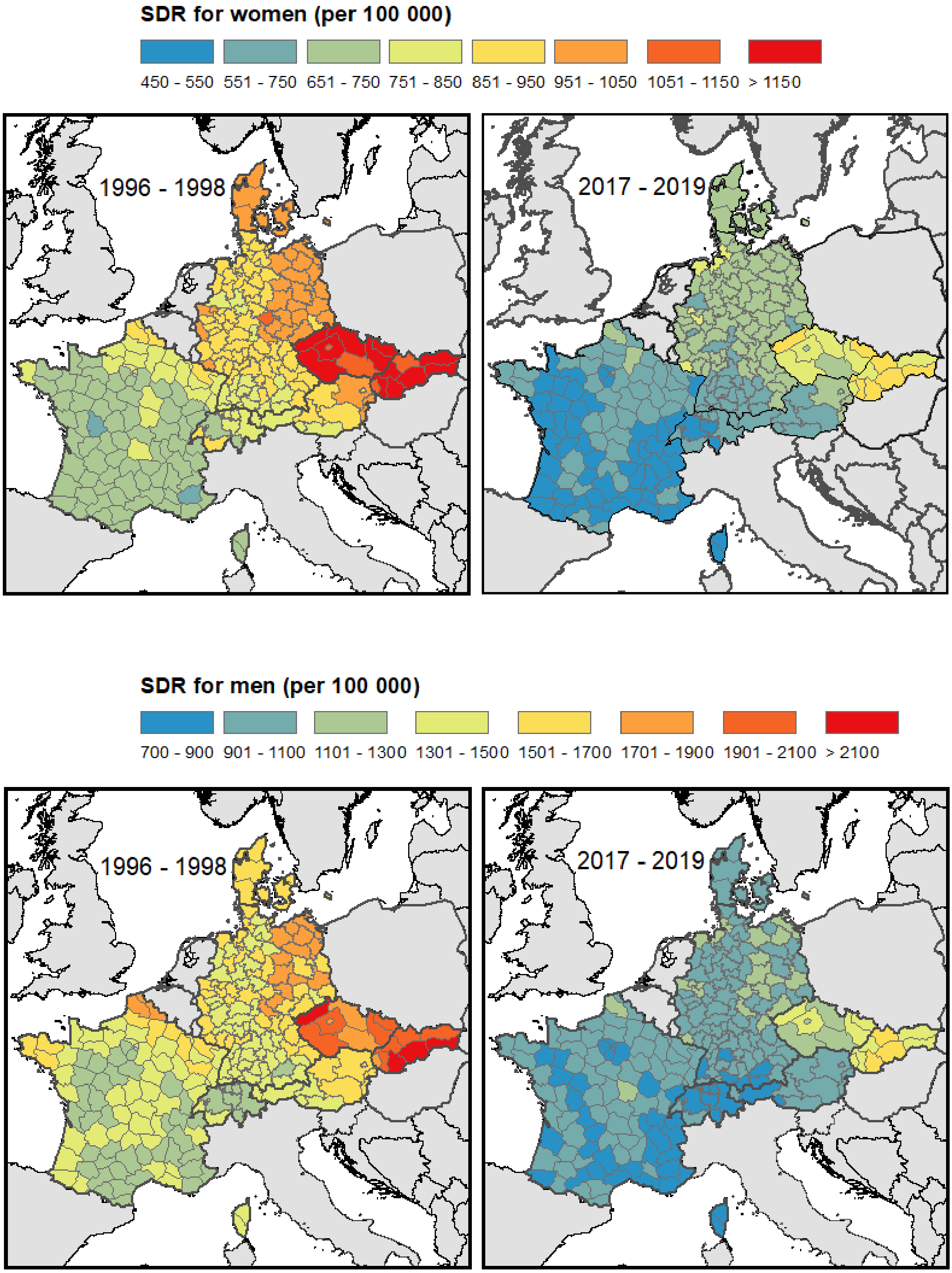

Figure S5 Standardised death rates in 1996–1998 and 2017–2019 in seven European countries for women and men separately; all ages

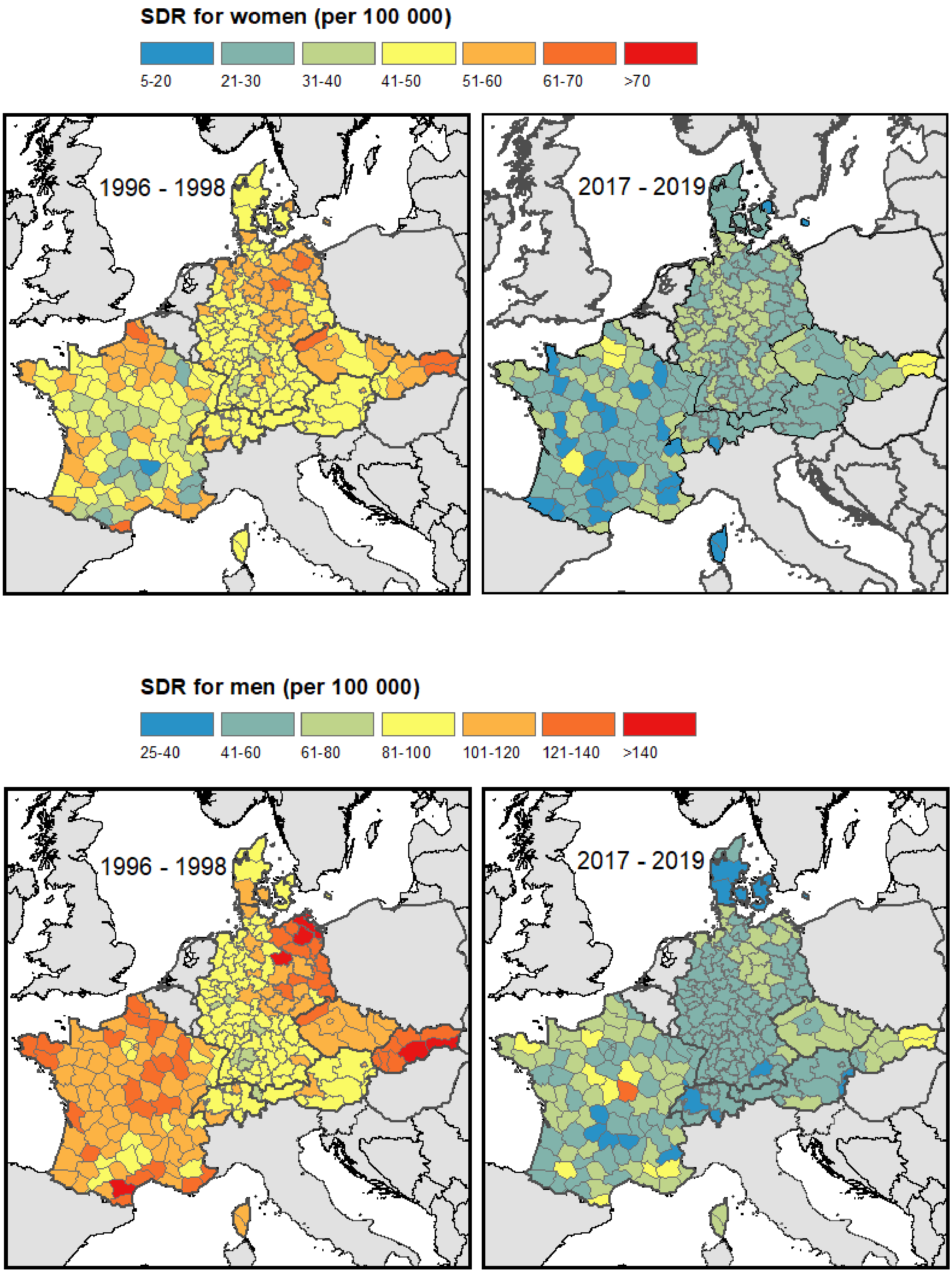

Figure S6 Standardised death rates in 1996–1998 and 2017–2019 in seven European countries for women and men separately; ages 0–39

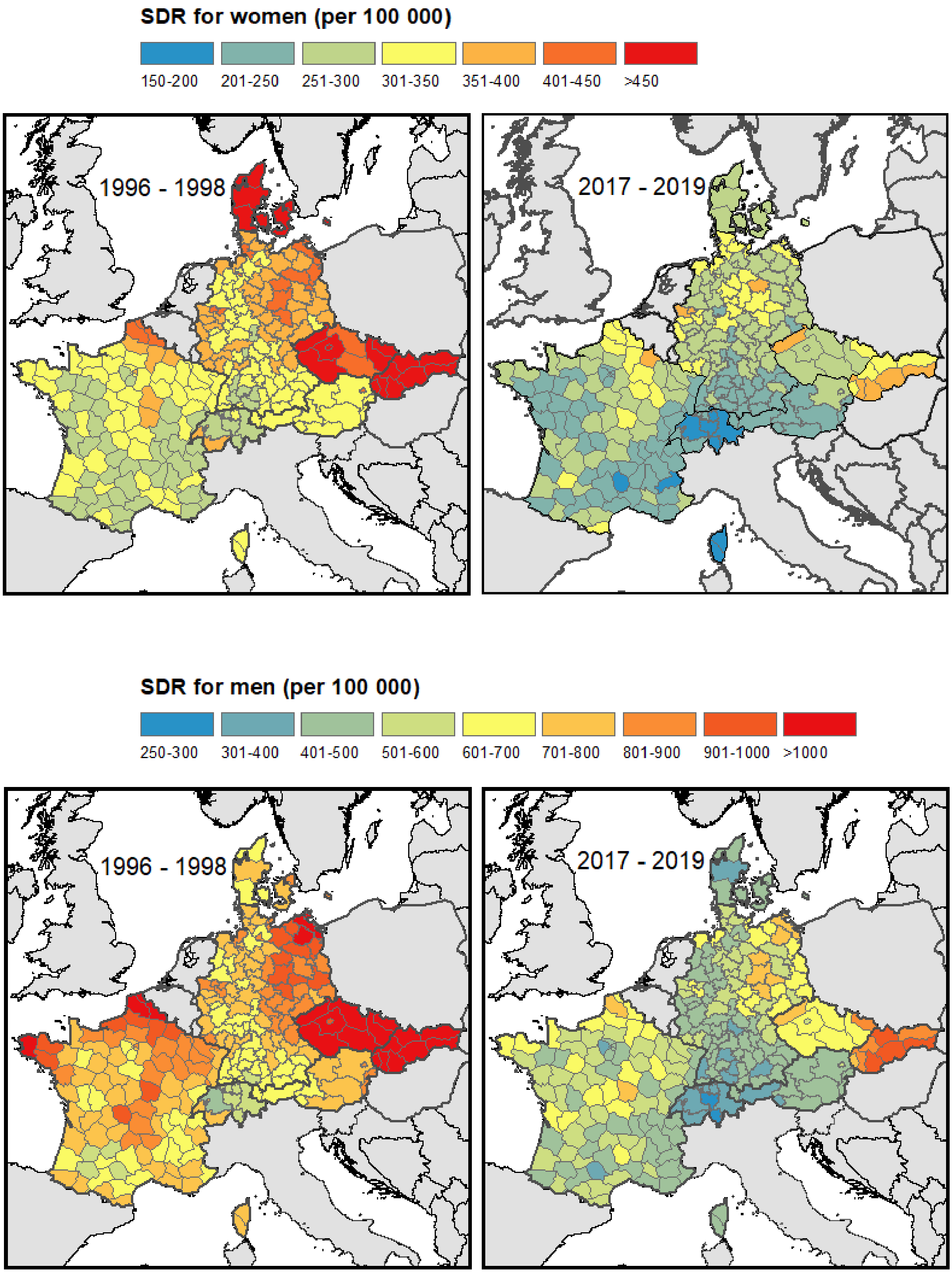

Figure S7 Standardised death rates in 1996–1998 and 2017–2019 in seven European countries for women and men separately; ages 40–64

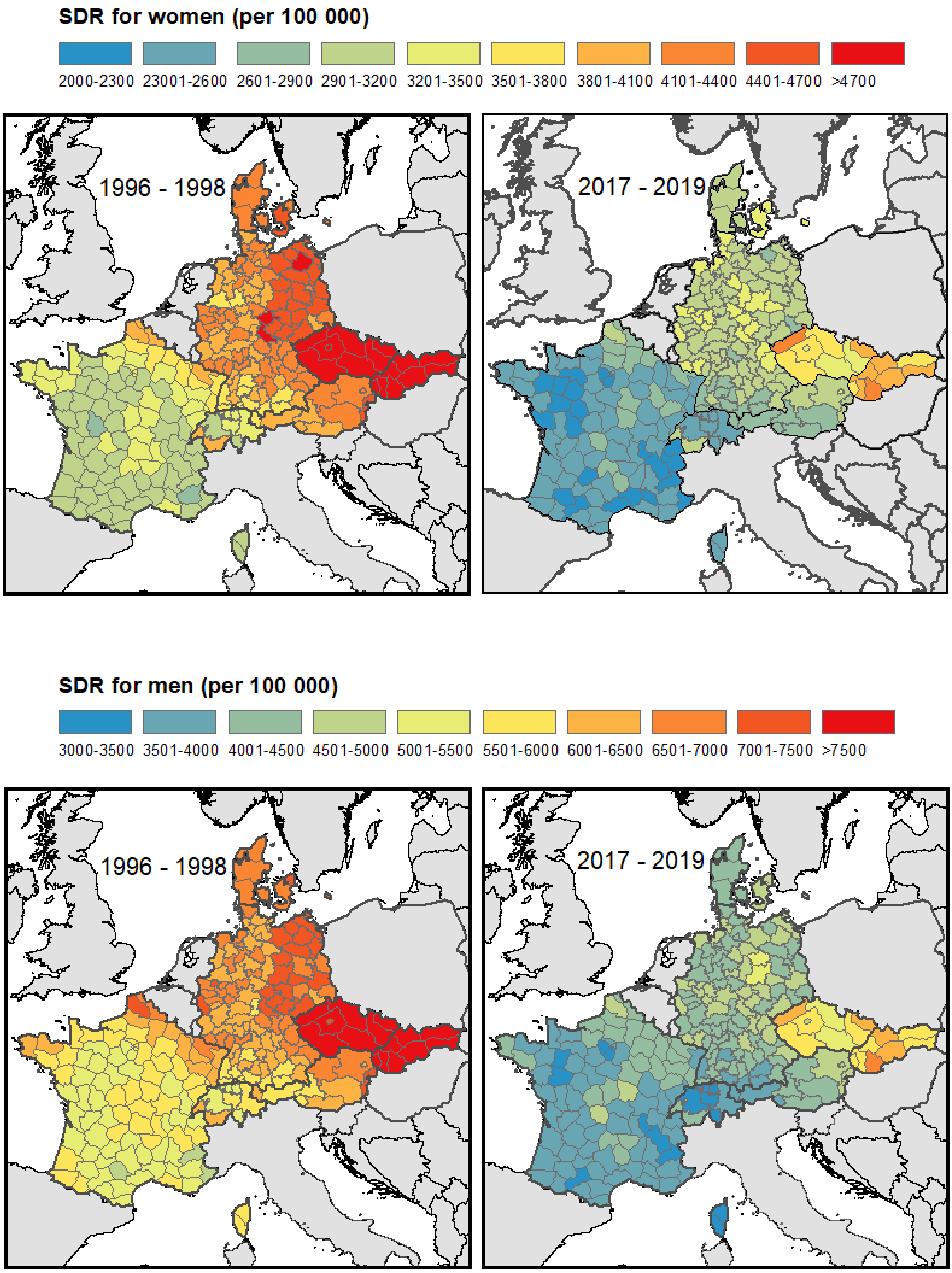

Figure S8 Standardised death rates in 1996–1998 and 2017–2019 in seven European countries for women and men separately; ages 65+

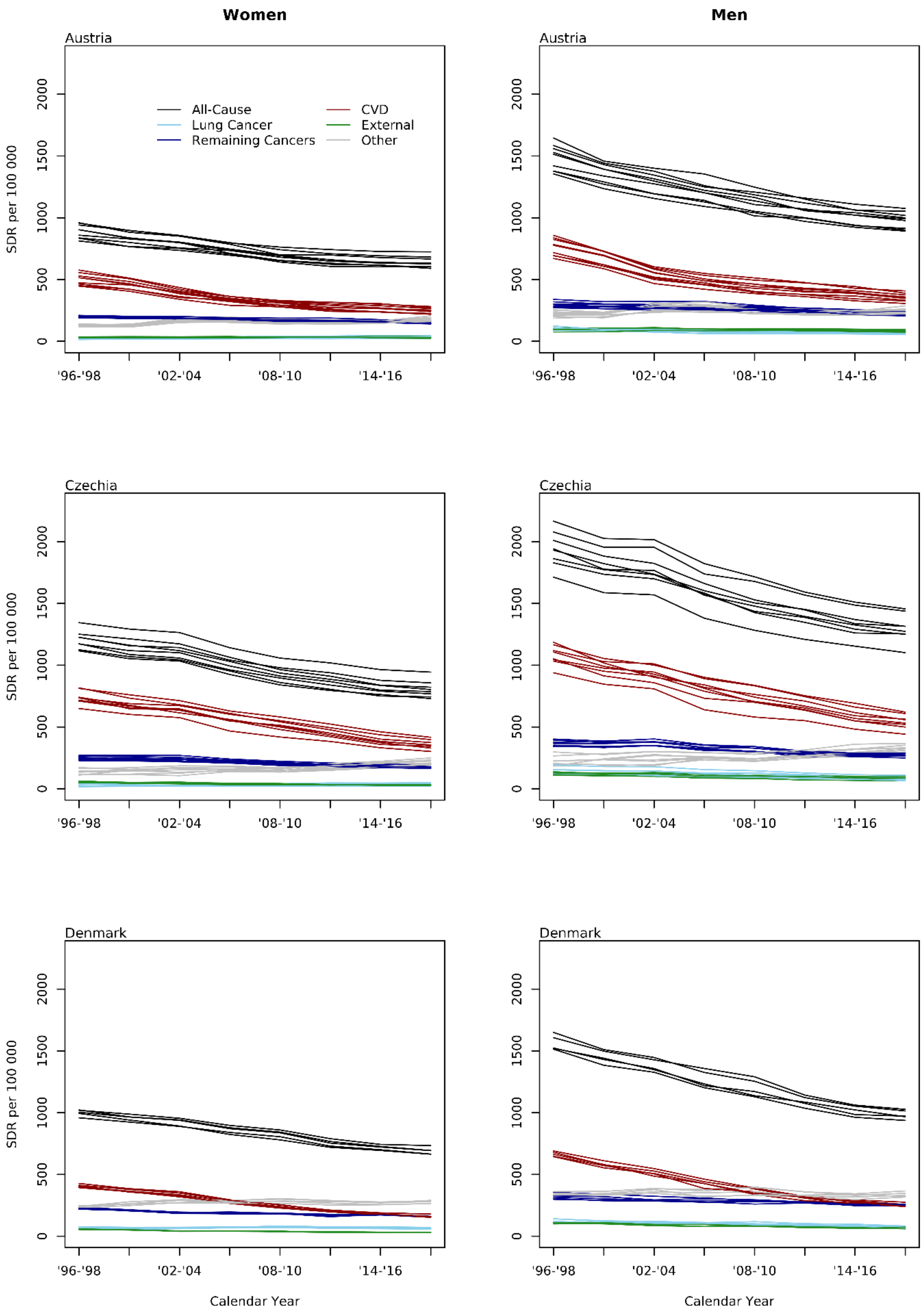

Figure S9 Standardised death rates in seven European countries by sex, cause of death and region, 1996/1998 to 2017/2019 *(to be continued on next page)*

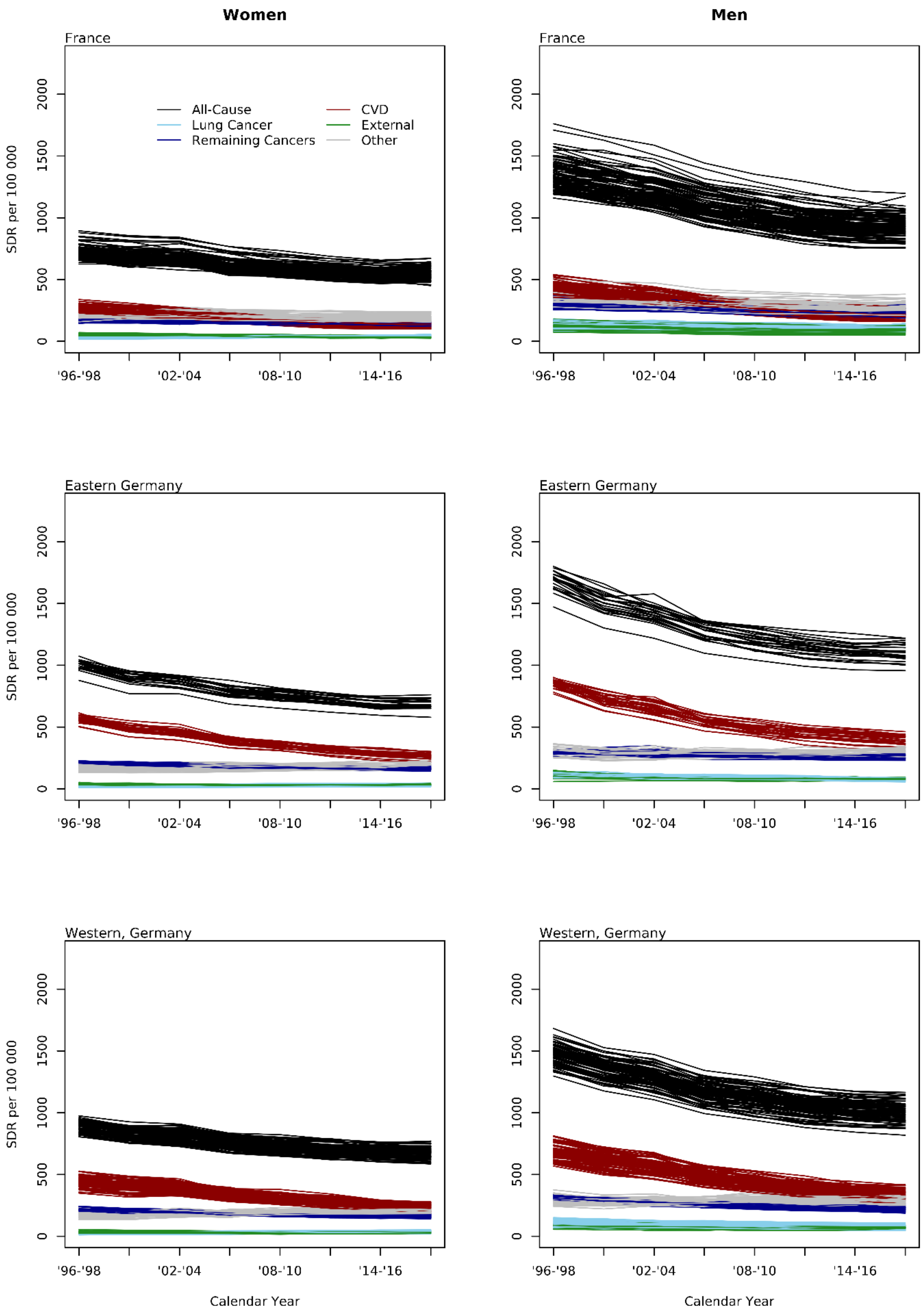

Figure S9 Standardised death rates in seven European countries by sex, cause of death and region, 1996/1998 to 2017/2019 *(continued)*
